# Supplementary material for: Effect of immunosuppressive drugs on glucose-stimulated insulin secretion: concentration-response studies in dynamic perifusion assays
Source: Front Immunol. 2026 Oct 5;17:1947013. doi: 10.3389/fimmu.2026.1947013 (PMC13677484; doi:10.3389/fimmu.2026.1947013)
Supplement: Supplementary file 1 [file DataSheet1.pdf]

frontiers

Supplementary Material

Effect of Immunosuppressive Drugs on Glucose-Stimulated Insulin Secretion: Concentration-Response Studies in Dynamic Perifusion Assays

Sung-Ting Chuang[1], Brandon Watts[1], Oscar Alcazar[1], and Peter Buchwald*[1,2]

[1]Diabetes Research Institute, Miller School of Medicine, University of Miami, Miami, Florida, USA;
[2]Department of Molecular and Cellular Pharmacology, Miller School of Medicine, University of Miami, Miami, Florida, USA

Supplementary Figures and Tables

- Supplementary Table S1. Checklist for reporting human islet preparations used in research.
- Supplementary Figure S1. Effect of immunosuppressive drugs at various concentrations on the viability of human islets.
- Supplementary Figure S2. Representative images illustrating the effects of immunosuppressive drugs at various concentrations on the viability of human islets.

1.1 Supplementary Tables

Table S1. Checklist for reporting human islet preparations used in research. Adapted from Hart NJ and Powers AC. *Diabetologia* (2019) 62:212. Data for the islet preparations used for the present dynamic perifusion GSIS studies.

**Islet preparation**	**1**	**2**	**3**	**4**	**5**
Unique identifier	44779828	45438526	48430443	43777471	44269935
Donor age (years)	58	25	50	57	29
Donor sex (M/F)	Female	Male	Male	Male	Male
Donor BMI (kg/m^2)	26.30	31.30	26.30	28.80	26.50
Donor HbA_{1c} or other measure of blood glucose control	5.5	5.4	5.2	5.9	5.4
Origin/source of islets[b]	IIDP	IIDP	IIDP	IIDP	IIDP
Islet isolation center	Wisconsin	Pennsylvania	Prodo Labs	Prodo Labs	Wisconsin
Donor history of diabetes? Please select yes/no from drop down list	No	No	No	No	No
Diabetes duration (years)					
Glucose-lowering therapy at time of death[c]					

Continues on the next page

Donor cause of death	Stroke	Anoxia	Head trauma	Stroke	Stroke
Warm ischemia time (h)	No	No	No	No	No
Cold ischemia time (h)	02:18	04:07	10:01	06:49	06:30
Estimated purity (%)	95	90	90	95	84
Estimated viability (%)	98	90	95	95	98
Total culture time (h)[d]	59	46	90	171	55
Glucose-stimulated insulin secretion or other functional measurement[e]	Glucose Stimulated Insulin Release Stimulation Index (SI): *1.6*	Glucose Stimulated Insulin Release Stimulation Index (SI): *2.8*	Glucose Stimulated Insulin Release Stimulation Index (SI): *2.7*	N/A	Glucose Stimulated Insulin Release Stimulation Index (SI): *5.0*
Handpicked to purity? Please select yes/no from drop down list	No	No	No	No	No
Additional notes					

[a]If you have used more than eight islet preparations, please complete additional forms as necessary

[b]For example, IIDP, ECIT, Alberta IsletCore

[c]Please specify the therapy/therapies

[d]Time of islet culture at the isolation center, during shipment and at the receiving laboratory

[e]Please specify the test and the results

**Islet preparation**	**6**	**7**	**8**	**9**	**10**
Unique identifier	46859494	47210981	47290391	43474066	48585017
Donor age (years)	23	43	54	54	50
Donor sex (M/F)	Male	Male	Female	Male	Male
Donor BMI (kg/m^2)	30.70	20.10	30.90	22.30	27.90
Donor HbA_{1c} or other measure of blood glucose control	5.6	5.6	4.7	5.3	4.6
Origin/source of islets[b]	IIDP	IIDP	IIDP	IIDP	IIDP
Islet isolation center	SC-ICRC	VCU	Arizona	SC-ICRC	Arizona
Donor history of diabetes? Please select yes/no from drop down list	No	No	No	No	No
Diabetes duration (years)					
Glucose-lowering therapy at time of death[c]					

Continues on the next page

Donor cause of death	Head trauma	Anoxia	Stroke	Stroke	Stroke
Warm ischemia time (h)	Yes	No	No	Yes	No
Cold ischemia time (h)	05:59	13:00	04:26	10:32	04:51
Estimated purity (%)	83	90	95	95	95
Estimated viability (%)	95	93	92	95	96
Total culture time (h)[d]	123	45	61	144	58
Glucose-stimulated insulin secretion or other functional measurement[e]	Glucose Stimulated Insulin Release Stimulation Index (SI): *1.9*	Glucose Stimulated Insulin Release Stimulation Index (SI): *3.4*	N/A	Glucose Stimulated Insulin Release Stimulation Index (SI): *1.5*	*N/A*
Handpicked to purity? Please select yes/no from drop down list	No	No	No	No	No
Additional notes					

[a]If you have used more than eight islet preparations, please complete additional forms as necessary

[b]For example, IIDP, ECIT, Alberta IsletCore

[c]Please specify the therapy/therapies

[d]Time of islet culture at the isolation center, during shipment and at the receiving laboratory

[e]Please specify the test and the results

**Islet preparation**	**11**	**12**	**13**	**14**	**15**
Unique identifier	50439434	52384438	56511686	51186627	55002262
Donor age (years)	21	26	54	45	53
Donor sex (M/F)	Male	Male	Female	Male	Male
Donor BMI (kg/m^2)	26.90	26.50	34.40	22.70	31.10
Donor HbA_{1c} or other measure of blood glucose control	5.1	5.2	4.9	5.4	5.4
Origin/source of islets[b]	IIDP	IIDP	IIDP	IIDP	IIDP
Islet isolation center	Pennsylvania	Wisconsin	Wisconsin	Wisconsin	SC-ICRC
Donor history of diabetes? Please select yes/no from drop down list	No	No	No	No	No
Diabetes duration (years)					
Glucose-lowering therapy at time of death[c]					

Continues on the next page

Donor cause of death	Anoxia	Anoxia	Stroke	Stroke	Anoxia
Warm ischemia time (h)	No	Yes	No	No	Yes
Cold ischemia time (h)	02:53	06:15	07:30	09:52	06:48
Estimated purity (%)	90	85	85	92	80
Estimated viability (%)	95	98	98	98	95
Total culture time (h)[d]	88	73	59	62	106
Glucose-stimulated insulin secretion or other functional measurement[e]	Glucose Stimulated Insulin Release Stimulation Index (SI): *4.4*	Glucose Stimulated Insulin Release Stimulation Index (SI): *3.6*	N/A	Glucose Stimulated Insulin Release Stimulation Index (SI): *5.3*	Glucose Stimulated Insulin Release Stimulation Index (SI): *2.9*
Handpicked to purity? Please select yes/no from drop down list	No	No	No	No	No
Additional notes					

[a]If you have used more than eight islet preparations, please complete additional forms as necessary

[b]For example, IIDP, ECIT, Alberta IsletCore

[c]Please specify the therapy/therapies

[d]Time of islet culture at the isolation center, during shipment and at the receiving laboratory

[e]Please specify the test and the results

**Islet preparation**	**16**	**17**	**18**	**19**	**20**
Unique identifier	49103743	62450160			
Donor age (years)	57	44			
Donor sex (M/F)	Female	Female			
Donor BMI (kg/m^2)	28.70	25.90			
Donor HbA_{1c} or other measure of blood glucose control	5.6	5.2			
Origin/source of islets[b]	IIDP	IIDP			
Islet isolation center	Prodo Labs	Wisconsin			
Donor history of diabetes? Please select yes/no from drop down list	No	No			
Diabetes duration (years)					
Glucose-lowering therapy at time of death[c]					

Continues on the next page

Donor cause of death	Stroke	Anoxia			
Warm ischemia time (h)	No	No			
Cold ischemia time (h)	09:01	08:00			
Estimated purity (%)	95	92			
Estimated viability (%)	95	98			
Total culture time (h)[d]	166	58			
Glucose-stimulated insulin secretion or other functional measurement[e]	Glucose Stimulated Insulin Release Stimulation Index (SI): *4.5*	N/A			
Handpicked to purity? Please select yes/no from drop down list	No	No			
Additional notes					

[a]If you have used more than eight islet preparations, please complete additional forms as necessary

[b]For example, IIDP, ECIT, Alberta IsletCore

[c]Please specify the therapy/therapies

[d]Time of islet culture at the isolation center, during shipment and at the receiving laboratory

[e]Please specify the test and the results

1.2 Supplementary Figures

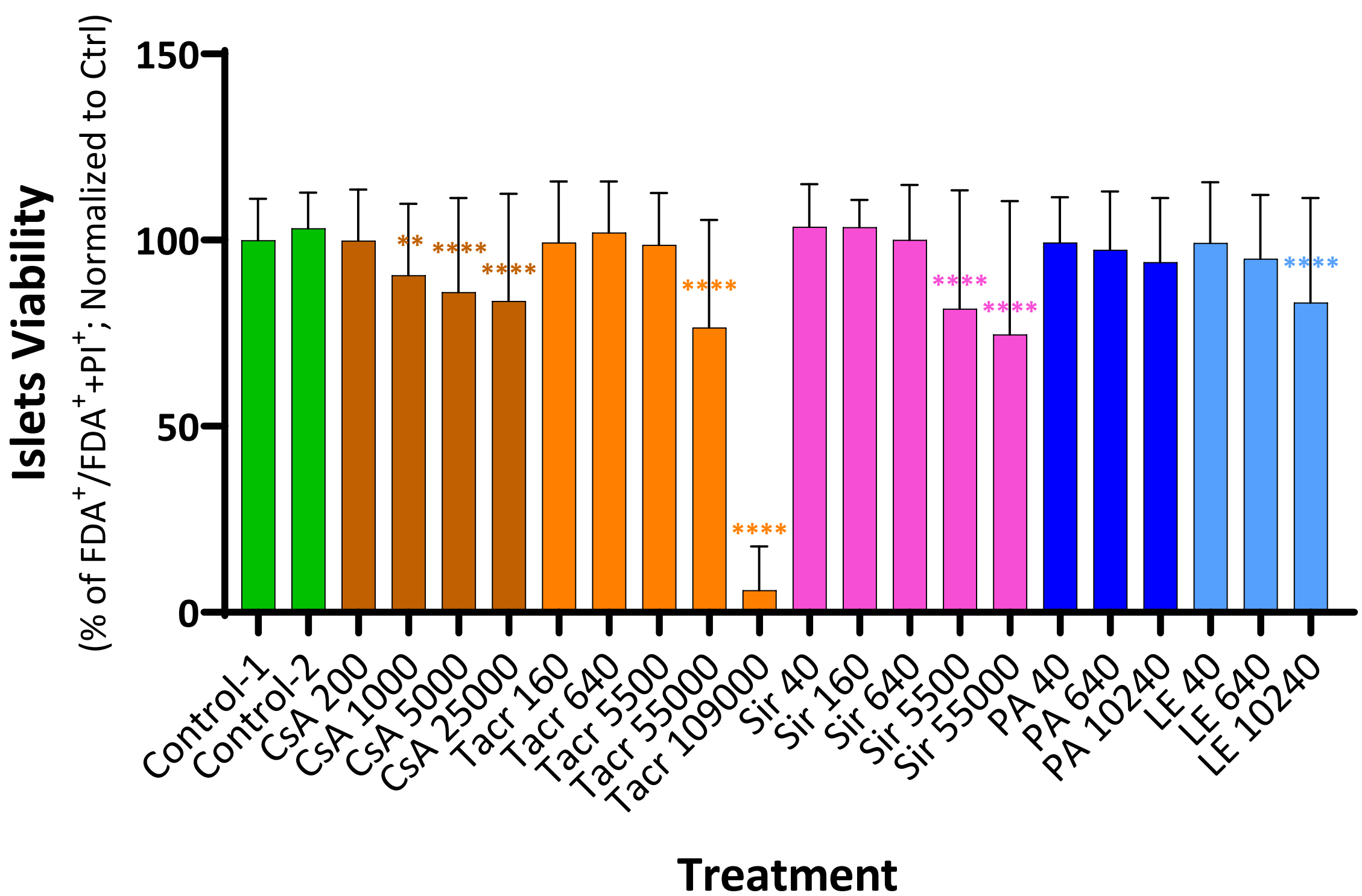

Figure S1. Effect of immunosuppressive drugs at various concentrations on the viability of human islets. Isolated human islets were exposed for 24 h to the drugs as shown (cyclosporine A, tacrolimus, sirolimus, prednisolone acetate, and loteprednol etabonate; concentrations in nM) and viability was estimated by staining with fluorescein diacetate (FDA) and propidium iodide (PI) dyes. Asterisks indicate statistically significant differences versus control as determined by one-way ANOVA followed by Dunnett's multiple comparison test (**: $p < 0.01$, ****: $p < 0.0001$).

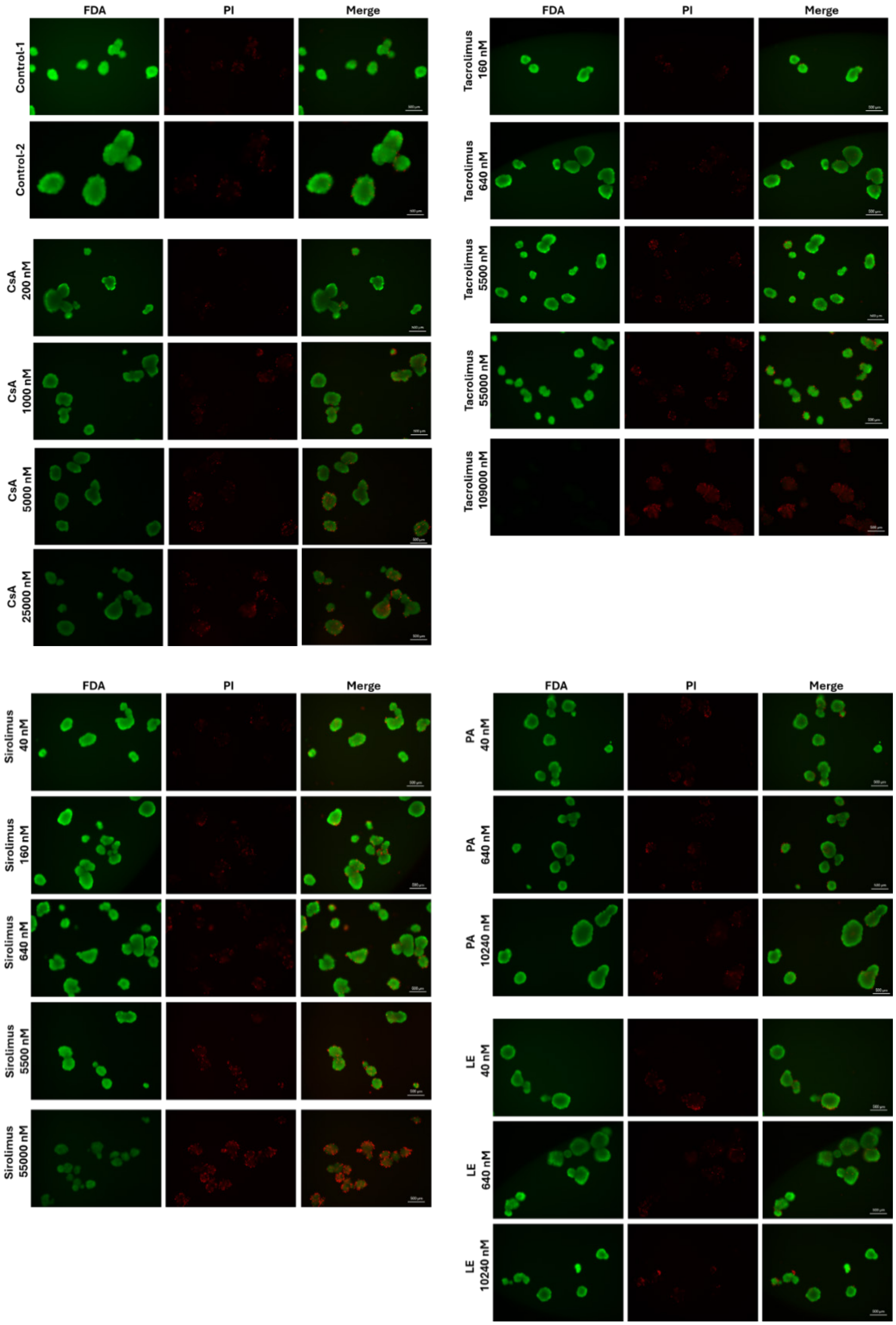

Figure S2. Representative images illustrating the effects of immunosuppressive drugs at various concentrations on the viability of human islets after 24 h exposure as revealed by staining with fluorescein diacetate (FDA) and propidium iodide (PI) dyes and quantified in Figure S1.
